# *N*-[3-(Dimethyl­amino)­prop­yl]-*N*′-(2-hy­droxy-5-methyl­phen­yl)oxamide

**DOI:** 10.1107/S1600536812007957

**Published:** 2012-02-29

**Authors:** Yong-Jun Zheng, Kang Zheng, Zhi-Yong Wu, Yantuan Li

**Affiliations:** a Marine Drug and Food Institute, Ocean University of China, Qingdao, Shandong 266003, People’s Republic of China; b Key Laboratory of Marine Drugs, Chinese Ministry of Education, Ocean University of China, Qingdao, People’s Republic of China

## Abstract

In the title compound, C_14_H_21_N_3_O_3_, the oxamide group has a *transoid* conformation. In the crystal, the mol­ecules are connected by N—H⋯O and O—H⋯N hydrogen bonds into a double chain running along the *b* axis.

## Related literature

For the use of *N*,*N*′-bis­(substituted)oxamides in the synthesis of nuclear complexes, see: Ojima & Nonoyama (1988 ▶); Ruiz *et al.* (1999 ▶). For related compounds, see: Han *et al.* (2007 ▶); Martinez *et al.* (1998 ▶); Yue *et al.* (2012 ▶). 
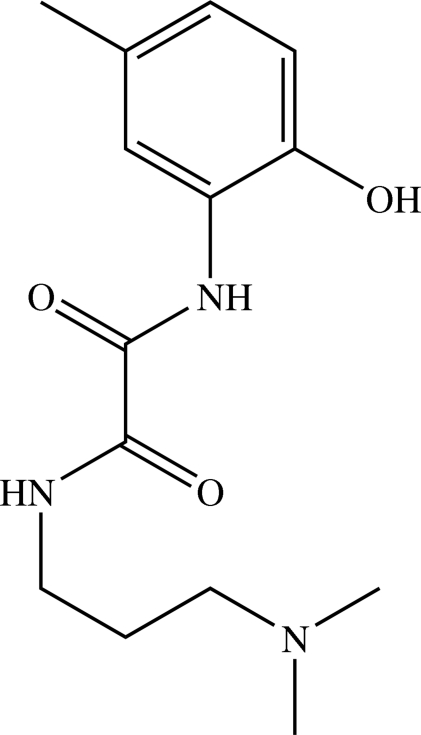


## Experimental

### 

#### Crystal data

C_14_H_21_N_3_O_3_
*M* *_r_* = 279.34
Monoclinic, 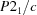

*a* = 11.542 (2) Å
*b* = 10.304 (2) Å
*c* = 13.860 (3) Å
β = 109.16 (3)°
*V* = 1557.2 (5) Å^3^
*Z* = 4
Mo *K*α radiation
μ = 0.09 mm^−1^
*T* = 296 K
0.27 × 0.24 × 0.17 mm

#### Data collection

Bruker APEX diffractometer
Absorption correction: multi-scan (*SADABS*; Sheldrick, 2003 ▶) *T*_min_ = 0.977, *T*_max_ = 0.986
7686 measured reflections
3099 independent reflections
2093 reflections with *I* > 2σ(*I*)
*R*_int_ = 0.022
Standard reflections: 0

#### Refinement

*R*[*F* ^2^ > 2σ(*F* ^2^)] = 0.053
*wR*(*F* ^2^) = 0.151
*S* = 1.02
3099 reflections
196 parameters
1 restraint
H atoms treated by a mixture of independent and constrained refinement
Δρ_max_ = 0.30 e Å^−3^
Δρ_min_ = −0.26 e Å^−3^

### 

Data collection: *SMART* (Bruker, 2002 ▶); cell refinement: *SAINT* (Bruker, 2002 ▶); data reduction: *SAINT*; program(s) used to solve structure: *SHELXS97* (Sheldrick, 2008 ▶); program(s) used to refine structure: *SHELXL97* (Sheldrick, 2008 ▶); molecular graphics: *XP* (Siemens, 1994 ▶); software used to prepare material for publication: *WinGX* (Farrugia, 1999 ▶).

## Supplementary Material

Crystal structure: contains datablock(s) I, global. DOI: 10.1107/S1600536812007957/bt5821sup1.cif

Structure factors: contains datablock(s) I. DOI: 10.1107/S1600536812007957/bt5821Isup2.hkl

Supplementary material file. DOI: 10.1107/S1600536812007957/bt5821Isup3.cml

Additional supplementary materials:  crystallographic information; 3D view; checkCIF report

## Figures and Tables

**Table 1:** Hydrogen-bond geometry (Å, °)

*D*—H⋯*A*	*D*—H	H⋯*A*	*D*⋯*A*	*D*—H⋯*A*
N2—H2⋯O2^i^	0.92 (3)	2.13 (3)	2.945 (2)	147 (2)
O1—H1*A*⋯N3^ii^	0.90 (2)	1.76 (2)	2.654 (2)	168 (3)

Symmetry codes: (i) 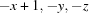; (ii) 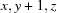.

**Fig. 1.**
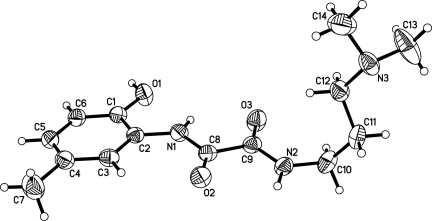
The molecular structure of the title compound. The displacement ellipsoids are drawn at the 30% probability levels and H atoms are shown as small spheres of arbitrary radii.

**Fig. 2.**
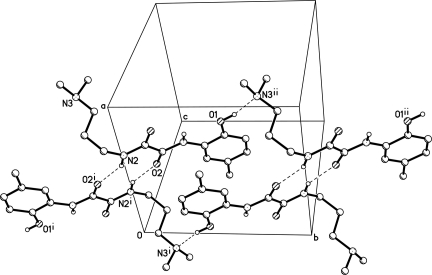
A view of a hydrogen bonding chain extending along the b-axis. [Symmetry codes: i = -x + 1, -y, -z; ii = x, y + 1, z.]

## supplementary crystallographic information

### Comment

*N*,*N*'-bis(substituented)oxamides as multiatom bridging ligands has played an important role in designing and synthesizing polymetallic systems (Ojima & Nonoyama, 1988; Ruiz *et al.*, 1999). Due to the difficulties of synthesis only few dissymmetrical bis-substituented-oxamide ligand has been reported. In order to provide more examples of such ligand, quite recently, we reported the structure of (3-{[*N*-(5-Chloro-2-hydroxyphenyl)oxamoyl]amino}propyl)dimethylazanium perchlorate (Yue *et al.*,2012). In continuation of our earlier work, the title compound was synthesized and its structure is reported here.

As shown in Fig. 1, the title compound has a *trans-*conformation of the oxamide group. The whole compound likes the alphabet `*L*'. The benzene ring is almost coplanar to the oxamide group [11.44 (9)°], just like that in the compound of *N*,*N*'-bis(2-Hydroxyphenyl)oxamide (Martinez *et al.*, 1998). However, the plane through the other substituent group, aminopropyl, is perpendicular to the oxamide plane [83.49 (12)°]. The torsion angle of C9—N2—C10—C11 is 106.7 (2)° (Table 1). And the conformation for the C10—C11 bond is *gauche*. While in the compound 2-(*N*'-[3-(Dimethylammonio)propyl)oxamido]benzoate (Han *et al.*, 2007), the corresponding angle is 151.3 (3)° and the conformation is anti.

A centrosymmetric dimer of a pair of the compounds is formed *via* the hydrogen bonds of the oxamide groups (Fig. 2, Table 2). These dimers are further assembled to a chain parallel to the *b-*axis through the hydrogen bonds between the phenolic hydroxyl groups and the tertiary amino groups.

### Experimental

All reagents were of AR grade and obtained commercially without further purification. The title compound was prepared according to the method proposed by Han *et al.* (2007). A tetrahydrofuran (THF) solution (8 ml) of ethyl oxalyl chloride (1.11 ml, 10 mmol) was added dropwise into a THF solution (50 ml) of 2-amino-4-methylphenol (1.23 g, 10 mmol) with continuous stirring. The mixture was stirred quickly for 1 h and became clear. Then 20 ml ethanol was further added and the mixture was added dropwise into the solution (10 ml) of 3-dimethylamino-propylamine (1.02 g, 10 mmol) with stirring and kept the temperature at 273 K for 9 h. The title compound was precipitated as a white powder and washed with ethanol for several times and dried under vacuum. Yield: 1.83 g (66%). Colourless crystals of compound with the suitable size for X-ray analysis were obtained from an ethanol/water (1:1) mixture by slow evaporation for one week at room temperature.

Anal. Calcd. for C_14_H_21_N_3_O_3_ (%): C, 60.20; H, 7.58; N, 15.04. Found: C, 60.33; H, 7.65; N, 15.00.

### Refinement

All H atoms were found in a difference Fourier map. Those bonded to N and O were freely refined with the O1—H1A bond restrained to a length of 0.86 (2) Å. Other H atoms were placed in calculated positions, with C—H = 0.93 (aromatic), 0.97 (methylene) and 0.96 (methyl), and refined using a riding model with *U*_iso_(H) = 1.2*U*_eq_C or 1.5*U*_eq_C(methyl).

### Crystal data

**Table:**

C_14_H_21_N_3_O_3_	*F*(000) = 600
*M**_r_* = 279.34	*D*_x_ = 1.191 Mg m^−^^3^
Monoclinic, *P*2_1_/*c*	Mo *K*α radiation, λ = 0.71073 Å
Hall symbol: -P 2ybc	Cell parameters from 2107 reflections
*a* = 11.542 (2) Å	θ = 2.5–23.3°
*b* = 10.304 (2) Å	µ = 0.09 mm^−^^1^
*c* = 13.860 (3) Å	*T* = 296 K
β = 109.16 (3)°	Block, colourless
*V* = 1557.2 (5) Å^3^	0.27 × 0.24 × 0.17 mm
*Z* = 4	

### Data collection

**Table:**

Bruker APEX diffractometer	3099 independent reflections
Radiation source: fine-focus sealed tube	2093 reflections with *I* > 2σ(*I*)
Graphite monochromator	*R*_int_ = 0.022
φ and ω scans	θ_max_ = 26.1°, θ_min_ = 2.5°
Absorption correction: multi-scan (*SADABS*; Sheldrick, 2003)	*h* = −6→14
*T*_min_ = 0.977, *T*_max_ = 0.986	*k* = −12→12
7686 measured reflections	*l* = −17→12

### Refinement

**Table:**

Refinement on *F*^2^	Primary atom site location: structure-invariant direct methods
Least-squares matrix: full	Secondary atom site location: difference Fourier map
*R*[*F*^2^ > 2σ(*F*^2^)] = 0.053	Hydrogen site location: inferred from neighbouring sites
*wR*(*F*^2^) = 0.151	H atoms treated by a mixture of independent and constrained refinement
*S* = 1.02	*w* = 1/[σ^2^(*F*_o_^2^) + (0.068*P*)^2^ + 0.3954*P*] where *P* = (*F*_o_^2^ + 2*F*_c_^2^)/3
3099 reflections	(Δ/σ)_max_ < 0.001
196 parameters	Δρ_max_ = 0.30 e Å^−^^3^
1 restraint	Δρ_min_ = −0.26 e Å^−^^3^

### Special details

**Table:**

Geometry. All e.s.d.'s (except the e.s.d. in the dihedral angle between two l.s. planes) are estimated using the full covariance matrix. The cell e.s.d.'s are taken into account individually in the estimation of e.s.d.'s in distances, angles and torsion angles; correlations between e.s.d.'s in cell parameters are only used when they are defined by crystal symmetry. An approximate (isotropic) treatment of cell e.s.d.'s is used for estimating e.s.d.'s involving l.s. planes.
Refinement. Refinement of *F*^2^ against ALL reflections. The weighted *R*-factor *wR* and goodness of fit *S* are based on *F*^2^, conventional *R*-factors *R* are based on *F*, with *F* set to zero for negative *F*^2^. The threshold expression of *F*^2^ > σ(*F*^2^) is used only for calculating *R*-factors(gt) *etc*. and is not relevant to the choice of reflections for refinement. *R*-factors based on *F*^2^ are statistically about twice as large as those based on *F*, and *R*-factors based on ALL data will be even larger.

### Fractional atomic coordinates and isotropic or equivalent isotropic displacement parameters (Å^2^)

**Table:**

	*x*	*y*	*z*	*U*_iso_*/*U*_eq_	
O1	0.78563 (15)	0.54467 (14)	0.12601 (15)	0.0756 (5)	
O2	0.52711 (14)	0.16017 (13)	0.05547 (12)	0.0675 (4)	
O3	0.81264 (15)	0.18892 (16)	0.01920 (14)	0.0812 (5)	
N1	0.66500 (17)	0.32727 (16)	0.08912 (13)	0.0551 (4)	
N2	0.67544 (19)	0.02499 (17)	−0.02835 (13)	0.0606 (5)	
N3	0.92876 (16)	−0.24706 (17)	0.16828 (17)	0.0720 (6)	
C1	0.67333 (18)	0.54785 (18)	0.13783 (15)	0.0535 (5)	
C2	0.60784 (17)	0.43098 (17)	0.12124 (14)	0.0491 (5)	
C3	0.49425 (19)	0.4244 (2)	0.13394 (15)	0.0574 (5)	
H3A	0.4521	0.3459	0.1235	0.069*	
C4	0.4419 (2)	0.5333 (2)	0.16214 (16)	0.0623 (5)	
C5	0.5060 (2)	0.6490 (2)	0.17547 (15)	0.0617 (6)	
H5	0.4715	0.7234	0.1927	0.074*	
C6	0.62038 (19)	0.65640 (19)	0.16368 (15)	0.0587 (5)	
H6	0.6618	0.7353	0.1733	0.070*	
C7	0.3173 (2)	0.5254 (3)	0.1756 (3)	0.0982 (9)	
H7A	0.3224	0.4715	0.2333	0.147*	
H7B	0.2593	0.4890	0.1152	0.147*	
H7C	0.2912	0.6109	0.1867	0.147*	
C8	0.62327 (18)	0.20947 (18)	0.05512 (14)	0.0522 (5)	
C9	0.7136 (2)	0.1393 (2)	0.01338 (15)	0.0568 (5)	
C10	0.7491 (3)	−0.0559 (2)	−0.07135 (18)	0.0756 (7)	
H10A	0.8154	−0.0044	−0.0798	0.091*	
H10B	0.6987	−0.0857	−0.1384	0.091*	
C11	0.8022 (2)	−0.1719 (2)	−0.00525 (19)	0.0739 (7)	
H11A	0.8523	−0.2210	−0.0365	0.089*	
H11B	0.7358	−0.2277	−0.0022	0.089*	
C12	0.8800 (2)	−0.1347 (2)	0.10290 (19)	0.0692 (6)	
H12A	0.9478	−0.0812	0.0999	0.083*	
H12B	0.8306	−0.0833	0.1333	0.083*	
C13	1.0343 (3)	−0.3023 (3)	0.1486 (4)	0.1352 (16)	
H13A	1.0639	−0.3754	0.1927	0.203*	
H13B	1.0979	−0.2381	0.1613	0.203*	
H13C	1.0112	−0.3299	0.0787	0.203*	
C14	0.9619 (3)	−0.2082 (3)	0.2779 (2)	0.1081 (10)	
H14A	1.0234	−0.1416	0.2926	0.162*	
H14B	0.9933	−0.2822	0.3206	0.162*	
H14C	0.8904	−0.1759	0.2908	0.162*	
H1	0.732 (2)	0.343 (2)	0.0811 (16)	0.062 (7)*	
H2	0.599 (2)	−0.003 (2)	−0.0308 (18)	0.078 (8)*	
H1A	0.824 (2)	0.622 (2)	0.139 (2)	0.109 (10)*	

### Atomic displacement parameters (Å^2^)

**Table:**

	*U*^11^	*U*^22^	*U*^33^	*U*^12^	*U*^13^	*U*^23^
O1	0.0618 (10)	0.0484 (9)	0.1234 (14)	−0.0114 (7)	0.0397 (10)	−0.0126 (9)
O2	0.0639 (9)	0.0513 (8)	0.0878 (11)	−0.0137 (7)	0.0256 (8)	−0.0038 (7)
O3	0.0699 (11)	0.0702 (10)	0.1088 (13)	−0.0180 (9)	0.0365 (10)	−0.0231 (9)
N1	0.0503 (10)	0.0458 (9)	0.0663 (11)	−0.0055 (8)	0.0153 (9)	−0.0007 (8)
N2	0.0644 (12)	0.0522 (10)	0.0612 (11)	−0.0056 (9)	0.0152 (9)	−0.0059 (8)
N3	0.0500 (10)	0.0522 (10)	0.1121 (16)	0.0014 (8)	0.0245 (10)	0.0097 (10)
C1	0.0525 (11)	0.0476 (11)	0.0571 (11)	−0.0021 (9)	0.0134 (9)	0.0028 (9)
C2	0.0503 (11)	0.0444 (10)	0.0477 (10)	−0.0003 (8)	0.0094 (8)	0.0034 (8)
C3	0.0544 (12)	0.0572 (12)	0.0576 (12)	−0.0071 (9)	0.0142 (10)	0.0030 (9)
C4	0.0556 (12)	0.0699 (14)	0.0602 (12)	0.0021 (11)	0.0176 (10)	0.0001 (10)
C5	0.0642 (13)	0.0608 (13)	0.0562 (12)	0.0106 (11)	0.0144 (10)	−0.0013 (10)
C6	0.0639 (13)	0.0459 (11)	0.0620 (12)	−0.0015 (9)	0.0147 (10)	−0.0016 (9)
C7	0.0717 (17)	0.106 (2)	0.128 (2)	−0.0018 (16)	0.0483 (17)	−0.0125 (19)
C8	0.0538 (12)	0.0433 (10)	0.0524 (11)	−0.0039 (9)	0.0078 (9)	0.0045 (8)
C9	0.0580 (12)	0.0503 (11)	0.0562 (11)	−0.0054 (10)	0.0109 (10)	0.0010 (9)
C10	0.1010 (19)	0.0682 (14)	0.0629 (14)	−0.0081 (13)	0.0340 (13)	−0.0143 (11)
C11	0.0895 (17)	0.0542 (13)	0.0863 (16)	−0.0011 (12)	0.0402 (14)	−0.0182 (12)
C12	0.0598 (13)	0.0476 (11)	0.0948 (17)	−0.0030 (10)	0.0182 (12)	−0.0023 (11)
C13	0.0772 (19)	0.096 (2)	0.253 (5)	0.0301 (17)	0.083 (3)	0.056 (3)
C14	0.097 (2)	0.096 (2)	0.100 (2)	0.0002 (17)	−0.0096 (17)	0.0116 (17)

### Geometric parameters (Å, º)

**Table:**

O1—C1	1.359 (3)	C5—H5	0.9300
O2—C8	1.222 (2)	C6—H6	0.9300
O3—C9	1.231 (2)	C7—H7A	0.9600
N1—C2	1.403 (3)	C7—H7B	0.9600
N1—C8	1.334 (2)	C7—H7C	0.9600
N2—C9	1.322 (3)	C8—C9	1.530 (3)
O1—H1A	0.904 (17)	C10—C11	1.509 (3)
N1—H1	0.83 (2)	C10—H10A	0.9700
N2—C10	1.451 (3)	C10—H10B	0.9700
N2—H2	0.92 (3)	C11—C12	1.522 (3)
N3—C13	1.450 (3)	C11—H11A	0.9700
N3—C12	1.464 (3)	C11—H11B	0.9700
N3—C14	1.494 (4)	C12—H12A	0.9700
C1—C6	1.377 (3)	C12—H12B	0.9700
C1—C2	1.400 (3)	C13—H13A	0.9600
C2—C3	1.381 (3)	C13—H13B	0.9600
C3—C4	1.390 (3)	C13—H13C	0.9600
C3—H3A	0.9300	C14—H14A	0.9600
C4—C5	1.383 (3)	C14—H14B	0.9600
C4—C7	1.511 (3)	C14—H14C	0.9600
C5—C6	1.385 (3)		
			
C1—O1—H1A	112.6 (18)	O2—C8—C9	122.52 (18)
C8—N1—C2	130.73 (19)	N1—C8—C9	110.60 (18)
C8—N1—H1	112.0 (15)	O3—C9—N2	124.6 (2)
C2—N1—H1	116.6 (15)	O3—C9—C8	120.83 (19)
C9—N2—C10	122.4 (2)	N2—C9—C8	114.58 (19)
C9—N2—H2	118.2 (16)	N2—C10—C11	112.48 (19)
C10—N2—H2	119.4 (16)	N2—C10—H10A	109.1
C13—N3—C12	111.7 (2)	C11—C10—H10A	109.1
C13—N3—C14	110.2 (3)	N2—C10—H10B	109.1
C12—N3—C14	109.6 (2)	C11—C10—H10B	109.1
O1—C1—C6	124.96 (18)	H10A—C10—H10B	107.8
O1—C1—C2	116.38 (17)	C10—C11—C12	112.92 (18)
C6—C1—C2	118.66 (19)	C10—C11—H11A	109.0
C3—C2—C1	120.35 (18)	C12—C11—H11A	109.0
C3—C2—N1	124.63 (17)	C10—C11—H11B	109.0
C1—C2—N1	114.99 (18)	C12—C11—H11B	109.0
C2—C3—C4	120.99 (19)	H11A—C11—H11B	107.8
C2—C3—H3A	119.5	N3—C12—C11	113.16 (18)
C4—C3—H3A	119.5	N3—C12—H12A	108.9
C5—C4—C3	118.1 (2)	C11—C12—H12A	108.9
C5—C4—C7	121.2 (2)	N3—C12—H12B	108.9
C3—C4—C7	120.7 (2)	C11—C12—H12B	108.9
C4—C5—C6	121.34 (19)	H12A—C12—H12B	107.8
C4—C5—H5	119.3	N3—C13—H13A	109.5
C6—C5—H5	119.3	N3—C13—H13B	109.5
C1—C6—C5	120.51 (19)	H13A—C13—H13B	109.5
C1—C6—H6	119.7	N3—C13—H13C	109.5
C5—C6—H6	119.7	H13A—C13—H13C	109.5
C4—C7—H7A	109.5	H13B—C13—H13C	109.5
C4—C7—H7B	109.5	N3—C14—H14A	109.5
H7A—C7—H7B	109.5	N3—C14—H14B	109.5
C4—C7—H7C	109.5	H14A—C14—H14B	109.5
H7A—C7—H7C	109.5	N3—C14—H14C	109.5
H7B—C7—H7C	109.5	H14A—C14—H14C	109.5
O2—C8—N1	126.9 (2)	H14B—C14—H14C	109.5
			
C8—N1—C2—C3	7.4 (3)	O1—C1—C6—C5	−178.97 (19)
C8—N1—C2—C1	−170.86 (19)	C2—C1—C6—C5	1.6 (3)
C9—N2—C10—C11	106.7 (2)	C4—C5—C6—C1	0.2 (3)
N2—C10—C11—C12	−57.1 (3)	C2—N1—C8—O2	−9.1 (3)
O1—C1—C2—C3	178.43 (18)	C2—N1—C8—C9	170.82 (19)
C6—C1—C2—C3	−2.1 (3)	C10—N2—C9—O3	0.8 (3)
O1—C1—C2—N1	−3.2 (3)	C10—N2—C9—C8	−179.59 (18)
C6—C1—C2—N1	176.24 (17)	O2—C8—C9—O3	−176.04 (19)
C1—C2—C3—C4	0.8 (3)	N1—C8—C9—O3	4.0 (3)
N1—C2—C3—C4	−177.40 (19)	O2—C8—C9—N2	4.3 (3)
C2—C3—C4—C5	1.1 (3)	N1—C8—C9—N2	−175.62 (17)
C2—C3—C4—C7	179.8 (2)	C13—N3—C12—C11	78.6 (3)
C3—C4—C5—C6	−1.6 (3)	C14—N3—C12—C11	−158.9 (2)
C7—C4—C5—C6	179.7 (2)	C10—C11—C12—N3	178.2 (2)

### Hydrogen-bond geometry (Å, º)

**Table:**

*D*—H···*A*	*D*—H	H···*A*	*D*···*A*	*D*—H···*A*
N2—H2···O2^i^	0.92 (3)	2.13 (3)	2.945 (2)	147 (2)
O1—H1*A*···N3^ii^	0.90 (2)	1.76 (2)	2.654 (2)	168 (3)

Symmetry codes: (i) −*x*+1, −*y*, −*z*; (ii) *x*, *y*+1, *z*.
